# The human microbiome and gut–liver axis in people living with HIV

**DOI:** 10.1007/s11904-023-00657-x

**Published:** 2023-05-02

**Authors:** Maria J. Duarte, Phyllis C. Tien, Ma Somsouk, Jennifer C. Price

**Affiliations:** 1 Division of Gastroenterology and Hepatology, Department of Medicine, University of California, San Francisco, CA USA; 2 Division of Infectious Diseases, Department of Medicine, University of California, San Francisco, CA USA; 3 Department of Veterans Affairs Medical Center, San Francisco, CA USA

**Keywords:** Gut–liver axis, Microbiome, Microbial translocation, Dysbiosis

## Abstract

**Purpose of Review:**

Chronic liver disease is a major cause of morbidity and mortality amongst people living with HIV (PLWH). Emerging data suggests that gut microbial translocation may play a role in driving and modulating liver disease, a bi-directional relationship termed the gut–liver axis. While it is recognized that PLWH have a high degree of dysbiosis and gut microbial translocation, little is known about the gut–liver axis in PLWH.

**Recent Findings:**

Recent studies have shown that microbial translocation can directly lead to hepatic inflammation, and have linked gut microbial signatures, dysbiosis, and translocation to liver disease in PLWH. Additionally, multiple trials have explored interventions targeting the microbiome in PLWH.

**Summary:**

Emerging research supports the interaction between the gut microbiome and liver disease in PLWH. This offers new opportunities to expand our understanding of the pathophysiology of liver disease in this population, as well as to explore possible clinical interventions.

## Introduction

The human microbiome is comprised of trillions of bacteria, viruses, fungi, protozoa, and archaea that live on skin, gut lumen, and other mucosal surfaces. The study of the composition, byproducts, and effects of the human microbiome has flourished in the last decade, and many possible relationships have emerged between it and the pathogenesis of various diseases [1]. The unique association between the liver and the gut, connected via the portal system, systemic circulation and the biliary system, termed the gut–liver axis, has led to significant interest in how the microbiome and its byproducts may impact liver disease.

Chronic liver disease is a leading cause of morbidity and mortality among people living with HIV (PLWH) [2–5]. Viral hepatitis has historically been the cause of most liver disease amongst PLWH. The development of highly effective hepatitis B virus (HBV) antiviral therapy and curative hepatitis C virus (HCV) therapy has shifted this landscape, though data suggests that despite HCV cure, underlying liver disease may persist in PLWH [6]. HIV infection has also been linked to non-alcoholic fatty liver disease (NAFLD), which is common and more severe amongst PLWH than those without HIV [7, 8]. The drivers of this disparity remain unknown, but liver disease in PLWH has been increasingly linked to the gut microbiome. Dysbiosis, or perturbations in the composition and function of the microbiome, is especially striking in the setting of HIV infection [9, 10]. This review outlines the existing literature on the gut–liver axis in PLWH, starting with the impact of HIV on the gut microbiome and integrity, the effects of dysbiosis on the liver, and finally potential therapeutic interventions targeting the microbiome in PLWH.

## HIV and the Gut

### HIV and Immune Dysregulation

**Fig. 1:**
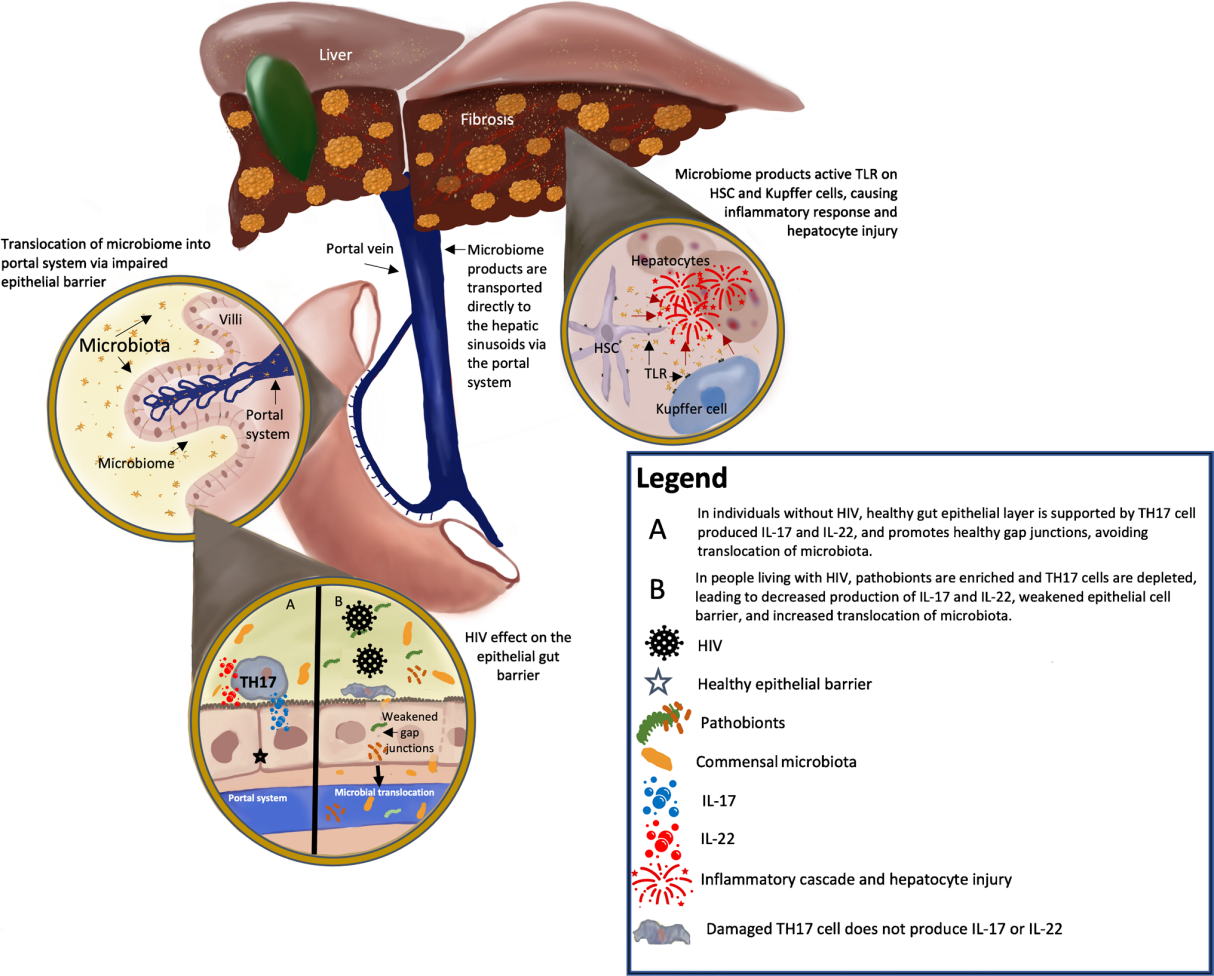
Microbial translocation and liver disease in PLWH

The human intestinal mucosa is comprised of a layer of columnar epithelial cells, lamina propria, and muscularis mucosa [11]. The epithelial layer plays an important role in maintaining a healthy barrier between the luminal contents and the circulatory system and is maintained by CD4 + TH17 + T cells in the lamina propria which produce IL-17 and IL-22 [11, 12]. These cytokines induce epithelial cell proliferation, epithelial tight junction formation, and the expression of claudins, mucin, and defensins (Fig. 1) [11].

Upon infection with HIV, there is a rapid loss of activated T cells in the gut. This is particularly pronounced in the lamina propria and is thought to be due to direct cytotoxic effects of the virus, as gut HIV RNA levels correlate with the amount of CD4 + cell loss. Mucosal TH17 + , TH22 + cells as well as other activated cytokine producing T cells are preferentially impacted and may show reduced functional capabilities [13–15]. The decrease in TH17 + cells leads to a reduction in IL-17 and IL-22, which in turn disrupts the epithelial cell barrier due to abnormalities in the IL-17-regulated tight junction protein occludin [16]. These tight junction breaches, as well as other consequences of decreased IL-17 and IL-22, increase the potential for bacterial translocation from the gut lumen into the portal and systemic circulation (Fig. 1) [17]. Murine models have corroborated this, showing that IL-17 loss leads to shifts in the microbiome composition with associated greater systemic inflammation and a weakened gut luminal barrier [18]. Studies evaluating macaque models infected with simian immunodeficiency virus (SIV) have directly visualized microbial translocation from the gut, and found that in the absence of gut translocation there is no systemic immune activation [17, 19].

Notably, these mucosal changes persist despite HIV treatment, that is, CD4 + cell loss in the gut was observed on histologic evaluation 6 months after initiating anti-retroviral therapy (ART) [14]. In chronic HIV infection, regulatory T cells (Treg) are upregulated and can help dampen immune activation; however, they have also been found to lead to collagen deposition in the gut mucosa, further depleting the CD4 + population in the gut [20]. Multiple studies have demonstrated disruption of the epithelial cell barrier in both acute and chronic HIV and SIV infection [19, 21, 22].

### HIV and Microbial Translocation

In a landmark study, Brenchley et al. provided a link between HIV infection, microbial translocation, and systemic immune activation [23]. In patients with chronic untreated HIV infection and in rhesus macaques with acute SIV infection, lipopolysaccharide (LPS), a marker of bacterial translocation, was elevated and strongly associated with levels of innate immune activation. ART reduced levels of LPS somewhat but did not normalize it [23]. Another study in macaque models showed that lack of histologic evidence for microbial translocation was associated with an absence of chronic immune activation [19]. Subsequent studies demonstrated that higher levels of soluble (s)CD14 (sCD14, a marker of monocyte response to LPS), gut epithelial barrier dysfunction, and innate immune activation predict mortality in patients with treated HIV infection [13, 24]. Taken together, these studies link the gut microbiome to chronic immune activation in PLWH gut barrier dysfunction and microbial translocation.

In addition to its effects on gut permeability, HIV infection may impact the composition of the microbiome. Vujkovic-Cvijin et al. demonstrated that the gut microbiomes from people living with untreated HIV were more likely to harbor pathogenic bacteria and less commensal bacteria compared to HIV-seronegative individuals [25]. Interestingly, the gut microbiota compositions of PLWH on ART varied widely, with some exhibiting a composition similar to those with untreated HIV and others closer to those without HIV; moreover, in PLWH on ART, the level of systemic inflammation was correlated to microbial composition patterning that resembled untreated HIV [25, 26]. Potentially harmful classes such as proteobacteria were enriched in HIV and commensal bacteria such as *Bacteroides* were depleted [25].

Finally, the bidirectionality between microbiome translocation and the gut luminal barrier in HIV was explored by Dillon et al. Their group demonstrated that HIV increases the amount of pathobionts in the gut microbiome, which can translocate across the weakened tight junctions in the epithelial barrier and activate innate immunity [10]. The activation of this innate immunity can in turn decrease TH17 + cells and lead to more epithelial barrier breakdown and thus microbial translocation [10]. More recent work has corroborated this and offered some optimism. A 2021 study analyzed markers of microbial translocation and immune activation in the peripheral serum of patients with treated HIV infection and suggested that ART leads to improvements in markers of microbial translocation and gut integrity after 2 years [27].

## The Human Microbiome and Liver Disease

### The Gut–Liver Axis

A strong physiologic link exists between the gut lumen and the liver, as all venous outflow from the intestinal tract flows through the portal system and into hepatic sinusoids. This connection is bidirectional, with bile acids produced by the liver flowing into the duodenal lumen via the common bile duct and the Sphincter of Oddi. In addition, the liver and the gut are connected to each other (and the rest of the body) via systemic circulation. As a result of this interconnection, the liver is perhaps the organ most directly exposed to the contents of the intestinal lumen, including microbial flora and its byproducts.

Indeed, microbial translocation directly into the liver has been studied in SIV models. Estes et al. visualized increased amounts of *E. coli* in hepatic tissue in macaques with SIV/AIDS and epithelial barrier dysfunction [19]. Over the years, the translocation of microbes and microbial byproducts across the intestinal epithelial barrier has been associated with the pro-inflammatory state driving liver diseases of diverse etiologies [28–30]. When microbial products translocate across the gut barrier, they enter the portal venous system and enter the hepatic sinusoids containing both Kupffer and hepatic stellate cells. Microbial associated molecular products (MAMPS) activate toll-like receptors on both Kupffer and stellate cells, leading to an inflammatory cascade mediated by Kupffer cells while stellate cell activation contributes to further injury and fibrosis (Fig. 1) [29].

As one example, the microbiome may play a significant role in the pathogenesis of NAFLD. In a groundbreaking experiment in 2013, Le Roy et al. demonstrated that the tendency to develop NAFLD in mice could be transmitted by transplanting gut microbiota [31]. While not specific to NAFLD, results of humans studies evaluating the effects of fecal microbiota transplant (FMT) suggest that the microbiome may mediate metabolic syndrome, since participants with metabolic syndrome who underwent intestinal infusion of gut microbiota from lean individuals had improved insulin [32]. While the exact mechanism by which the microbiome induces NAFLD is incompletely elucidated, steatohepatitis may be linked to certain microbial signatures, particularly a predominance of *Bacteroides* and *Ruminococcus* species [33]. Immunologic responses to these microbiota via toll-like receptors may play a role, as TLR4 deficiency in murine models has been associated with an attenuation in steatohepatitis [34]. Other microbiome factors implicated in the pathogenesis of NAFLD include microbiota-driven changes in caloric absorption, altered choline metabolism mimicking choline deficiency, and an increase in short chain fatty acids [35]. NAFLD is just one of many chronic liver conditions being increasingly linked to dysbiosis [36]. Moreover, different liver disease etiologies are associated with unique patterns of dysbiosis, with alcohol-associated liver disease having a higher predominance of *Enterobacteriaceae* as well as higher gut permeability when compared to other causes of cirrhosis [28, 37].

Cirrhosis itself also causes a profound dysbiosis, and the gut microbiome in patients with cirrhosis is enriched in *Enterobacteriaceae*, *Enterococcaceae*, and *Staphylococceae* as opposed to the predominantly *Bacteroides* and *Firmicutes* present in healthy individuals [28, 36, 38–41]. Emerging data suggest that microbiome composition may influence infectious outcomes in decompensated cirrhosis [42] that microbial products may be linked to the development of hepatocellular carcinoma, and that the cirrhotic liver may be less able to clear microbial byproducts [43]. Impaired clearance of microbial byproducts could then lead to greater immune activation, resulting in a bidirectional effect similar to microbial translocation in HIV. Our developing understanding of the relationship between the microbiome and liver disease can help guide diagnostics and surveillance of liver disease, with one study demonstrating that metagenomic microbiome signatures can predict non-alcoholic steatohepatitis (NASH) cirrhosis with an AUC of 0.91 [44], and another showing that microbiome analysis can identify early hepatocellular carcinoma [45].

### HIV, HCV, and the Gut Microbiome

There is a rich new field investigating the connection between the gut–liver axis an HIV infection. Animal studies evaluating macaques infected with SIV demonstrated a 20-fold increase of bacterial products in the livers of SIV-infected animals resulting in CXCL16 production by myeloid dendritic cells (mDCs) [46]. Hepatic mDC activation and recruitment of NK cells expressing CXCL16 receptor correlated significantly with liver damage and fibrosis [74]. In a different SIV model, dysbiosis in SIV infection persisted despite treatment with ART and was characterized by an increase in atypical mycobacteria, which in turn were shown to directly stimulate an inflammatory response in hepatocytes [47].

Most of the data surrounding the gut liver axis in PLWH focuses on co-infection with HCV, as PLWH and HCV experience more rapid progression of liver disease and fibrosis than those without HIV, and liver injury may persist in PLWH despite HCV cure [6, 48, 49]. Balagopal and colleagues first linked dysbiosis and advanced liver disease in PLWH and HCV, finding that elevated levels of LPS and other markers of microbial translocation were independently associated with cirrhosis in patients with both HIV and HCV [50]. Subsequent studies demonstrated that HIV and HCV were associated with a Kupffer cell–mediated inflammatory response in the liver [30, 51]. Marchetti et al. found that levels of the macrophage activation marker sCD14 correlated to severity of liver disease and predicted response to HCV treatment in PLWH [52].

One more example of how the pathobionts, microbiota perturbations, and immune activation in HIV can exacerbate liver disease lies in the tryptophan catabolism pathway. Tryptophan is an essential amino acid that is primarily catabolized via the kynurenine pathway, yielding metabolic byproducts including kynurenine. Both tryptophan and kynurenine levels can be measured in the serum, and elevations in the kynurenine to tryptophan (K:T) ratio are associated with increased tryptophan catabolism. Kynurenine binds to T cells and inhibits differentiation of TH17 + cells, thus leading to a reduction of IL-17 and IL-22 production which results in a disruption of localization of the TH17-regulated tight junction protein occludin [53]. This decreases in the integrity of tight junctions in the epithelial barrier, thus promoting translocation of microbes and their byproducts across the gut-mucosal wall, as well as greater immune activation [25, 54]. Microbiota enriched in the setting of HIV encode for greater numbers of tryptophan catabolism enzymes and thus increase the amount of kynurenine in the gut [25]. This leads to a bidirectional positive feedback cycle whereby the dysbiosis in HIV induces a higher K:T, which in turn prompts TH17 + cell loss, greater gut permeability, and greater dysbiosis [25, 55]. Elevated K:T has been associated with disease progression in HIV, as well as increased systemic inflammation and mortality [13, 56]. In the context of liver disease, Kardashian et al. found that a higher K:T ratio was associated with increased hepatic fibrosis in women living with HIV (with or without HCV coinfection) but not in women without HIV, suggesting that the altered gut microbiome in the setting of HIV might increase tryptophan catabolism and thus immune activation and liver fibrosis [57].

Data suggests that this bidirectionality may be partly the progression of liver disease amongst PLWH. In a study with over 600 participants that examined the contribution of microbial translocation and liver fibrosis to the immune activation marker sCD14, Reid et al. found microbial translocation contributed to an increased sCD14 level during HIV infection, whereas liver fibrosis played a stronger role during HCV mono-infection. Co-infected persons may be at greatest risk for progression, because of the independent effects of microbial translocation and liver fibrosis on immune activation that arise as a result of HIV [58]. Overall, these studies support the hypothesis that microbial translocation and the resulting inflammatory response contributes to liver disease in PLWH and is further exacerbated by the presence of HCV [52, 57–59].

### HIV, NAFLD, and the Gut Microbiome

With the advent of curative therapy for HCV, the epidemiology of liver disease in PLWH has shifted toward fatty liver. However, the role of gut microbiome in driving non-viral liver disease in PLWH is unclear [3, 7, 60–63]. HIV confers a higher risk of NAFLD as well as progression to NASH, fibrosis, and cirrhosis. In PLWH without viral hepatitis and with established NAFLD or elevated liver enzymes, the estimated prevalence of NASH is 42%, and ≥ F2 fibrosis is 22%, which may be higher than in persons without HIV (25% and 19%, respectively) [64, 65]. As a result, NASH is recognized as a rising cause of morbidity and mortality in PLWH; however, the drivers of this elevated risk remain unknown. As above, the microbiome has been shown to impact fatty liver disease in populations without HIV. More recent data suggests that the gut–liver axis and microbiome could be contributory to fatty liver disease progression in PLWH as well. Indeed, a recent pilot study found that certain microbiome signatures are associated with liver steatosis and fibrosis in PLWH [66••], suggesting a relationship between the gut and liver in HIV-related NAFLD. Further research is needed to further elucidate these interactions.

## Overview of Microbiome—Targeting Interventions

**Fig. 2:**
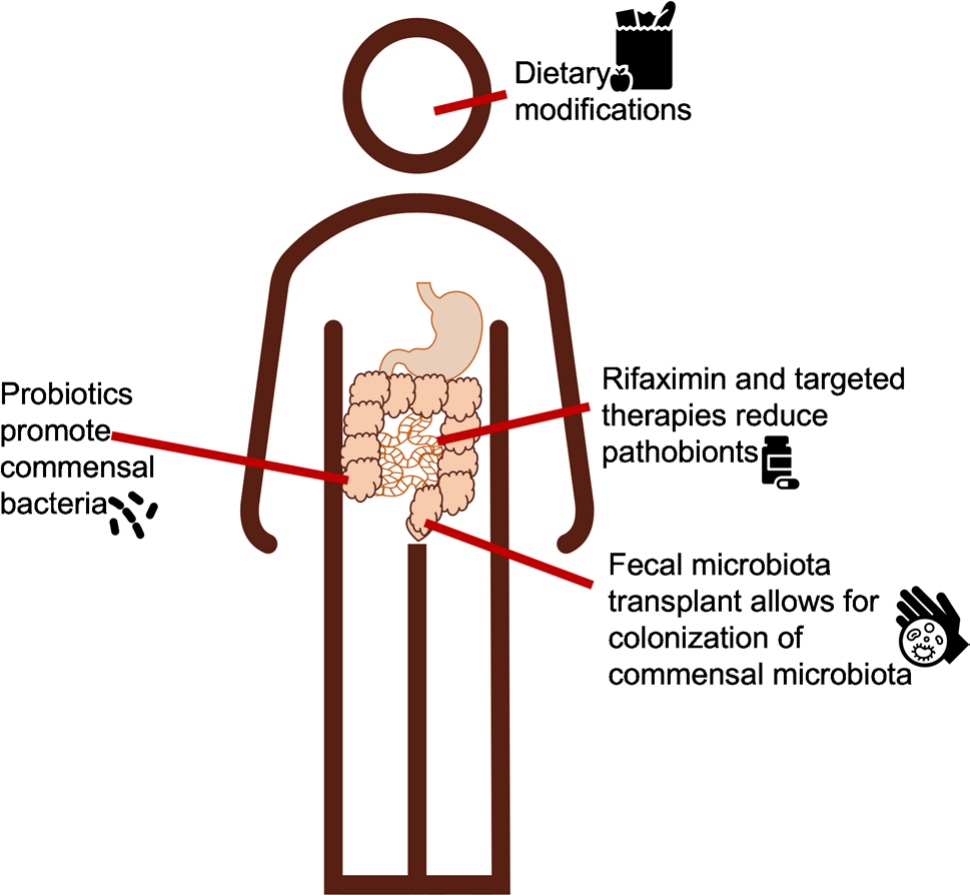
Microbial targeting therapies in PLWH

As gut dysbiosis has consistently been associated with increased inflammation, gut permeability, and poor outcomes in PLWH, numerous studies have investigated how the microbiome can be manipulated to improve health in this population. Interventions, including FMT (both in liquid and pill formulation), dietary modifications, and pathobiont removal parallel interventions in HIV-seronegative populations attempting to alter the dysbiosis in the gut to improve health outcomes [67]. Although a careful analysis of studies in people without HIV is outside the scope of this review, we highlight below how the microbiome has been targeted to improve liver outcomes in HIV seronegative populations (Fig. 2). While unfortunately no existing studies have focused on the microbiome as a therapeutic target to improve liver disease in PLWH, the data from other populations may allow for some extrapolation.

### Types of Interventions

One of the most well-known and promising interventions in the gut microbiome is fecal microbiota transplant (FMT), the inoculation of healthy diverse microbiota into people experiencing disease due to dysbiosis. FMT’s most notable success is in recurrent *Clostridium difficile* infection, which is caused by an overabundance of the toxin-producing bacteria *C. diff *[68]*.* The microbiome disequilibrium that allows *C. diff* to flourish and cause clinical infection is often brought about by the eradication of commensal bacteria with antibiotic use [68]. FMT allows inoculation of the gut lumen with a diverse, healthy microbiome community which (assuming appropriate microbial engraftment in the gut) is then able to out-compete the *C. diff *[69]. Further studies evaluating the role of antibiotics and other factors to increase engraftment with FMT are underway, and multiple companies now offer oral alternatives to the traditional endoscopy-delivered FMT [70]. Other interventions targeting the microbiome have focused on dietary modifications, including eliminating animal protein or the intake of probiotics, which contain commensal bacteria to promote colonization of the gut microbiome with healthy communities of microbiota. Additional strategies aimed at modifying the gut flora include therapies aimed at removing pathobionts with gut specific antibiotics such as Rifaxmin [71], which has already been shown to be effective at modulating small intestinal bacterial overgrowth (SIBO) and promoting healthy re-colonization of microbiota after traveler’s diarrhea [72]. Other more targeted approaches including the use of bacteriophages to target specific microbes and the use of chemical compounds to eliminate harmful microbial metabolic products and enzymes are also being explored [73].

### Microbiome Targeting Therapies for PLWH

Lessons learned from treating *C. diff* infection may be useful for manipulating the dysbiosis seen in PLWH. In a 2017 study, 6 PLWH underwent FMT in an attempt to modulate the gut microbiome (Table 1) [74]. However, unlike patients treated for *C. diff* infection, no significant changes in their gut microbiome compositions were observed post-FMT [74]. Recent studies involving FMT have yielded more encouraging results: one trial of FMT in 30 participants with HIV showed an attenuation in a biomarker of gut permability [75•], and a smaller study of weekly FMTs in 6 PWH demonstrated increased microbial diversity during the 6-week treatment period, with one subject experiencing improvement in biomarkers of gut permeability and inflammation [76•]. Despite these promising results, there are inherent risks to FMT—namely transplanting untested pathogenic bacteria—and caution is advised in trials involving immunocompromised populations. In 2019, a case report was published describing two patient deaths from sepsis after being inoculated with drug-resistant bacteria during FMT [77]. Both patients were immunocompromised. These cases sparked an FDA safety alert surrounding FMT as well as new protocols surrounding the screening of donor stool. Overall, more data are needed to determine whether FMT is a safe and viable option for the gut dysbiosis observed in HIV and whether it offers any mitigating effects on immune activation and long-term clinical outcomes.

**Table 1:** Literature evaluating microbiome targeting therapies for PLWH

Study (year)	*N*	Study design	Population	Intervention	Findings
Vujkovic-Cvijin et al. (2017) [74]	6	Pilot study	PLWH	FMT	No significant changes in stool microbiota composition or inflammatory markers in the peripheral blood after FMT
Serrano-Villar (2021) [75•]	14	Pilot placebo controlled trial	PLWH	FMT	Improved circulating serum levels of I-FABP, a marker of intestinal damage that independently predicts mortality
Utay et al. (2020) [76•]	6	Pilot study	PLWH	FMT	Receiving weekly FMT for 6 weeks led to increased microbial diversity during the treatment period. Microbial diversity returned to baseline afterward
Dillon et al. (2014) [78]	18	Cross sectional study	PLWH	Plant-based diet	Increased in Bacteroides on colon biopsy in PLWH who had a plant based diet
Noguera-Julian et al. (2016) [79]	240	Cross sectional study	PLWH	Plant-based diet	No significant differences in microbiota composition of stool samples in PLWH who consumed a plant based diet
Kristensen et al. (2016) [80]	315 total	Systematic review of 7 RCTs on probiotics	HIV seronegative populations	Probiotics	Overall no significant changes in microbiota composition of stool samples with administration of probiotic
Irvine et al. (2011) [81]	85	Cross sectional study	PLWH	Probiotics	PLWH who consumed probiotic yogurt reported improved GI symptoms
Villar-Garcia et al. (2015) [82]	44	Double blind randomized placebo controlled trial	PLWH	Probiotics	Probiotic use was associated with decreased markers of gut permeability (LBP) and IL6
d’Ettorre et al. (2017) [83]	10	Sub-study in a longitudinal pilot study	PLWH	Probiotics	Improved barrier integrity on ileal and colonic biopsies with increased TH17 levels
Williams et al. (2019) [71]	31	Sub-analysis within RCT	PLWH who are ART non-responders	Rifaximin	Rifaximin had no impact on microbiota composition on rectal swab
Tenorio et al. (2015) [84]	43	Sub-analysis within an RCT	PLWH who are ART non-responders	Rifaximin	No impact on serum markers of microbial translocation (LPS, sCD14) or CD8 cell activation on flow cytometry

Dietary interventions have also been studied in PLWH. In HIV-seronegative individuals, a plant-based diet has been associated with an enrichment of commensal bacterial species, including *Bacteroides *[78]*.* However, while HIV is associated with a depletion in *Bacteroides*, studies have thus far have failed to show an improvement in this dysbiosis with dietary modifications or plant-based diets [79]. A more promising dietary adjustment is the use of probiotics. Although the data for probiotic use in PLWH have been mixed (Table 1), there appears to be an improvement in GI symptoms [81]. In addition, in a randomized controlled trial in 2015, Villar-Garcia et al. demonstrated that probiotic use was associated with decreased lipopolysaccharide binding protein (LBP, a marker of translocation), as well as a decrease in IL-6 [82, 83]. Another study found that PLWH who took probiotics were more likely to have increases in TH17 + cells and improved gut epithelial barrier integrity [85].

Rifaximin has been raised as a possible drug to help modulate the dysbiosis and immune activation seen in HIV; however, thus far, data has failed to demonstrate an improvement in microbiome composition in PLWH taking rifaximin or in ART non-responders [71].

### Microbiome-Targeting Therapies for Liver Disease

In HIV seronegative populations, several of the above strategies have also been evaluated as potential therapies for chronic liver disease. In a murine model of alcohol-associated liver disease, FMT from alcohol “resistant” mice (who did not develop liver injury from alcohol) was shown to protect alcohol “sensitive” mice from alcohol-induced liver injury [86]. Similarly, FMT attenuates acute liver injury in mice by regulating cytokine balance, and FMT from humans resistant or sensitive to alcohol can modulate the hepatic sensitivity of mice to alcohol [87•, 88]. In a 2020 review evaluating 6 human studies using FMT to treat alcohol associated liver disease, all trials showed improvements in liver function and markers of liver injury, and three demonstrated a mortality benefit from FMT [89]. FMT may also improve antibiotic resistance patterns in cirrhosis [90], decrease HBeAg levels in HBV [91], improve hepatic encephalopathy outcomes [92], and improve liver enzymes in patients with primary sclerosing cholangitis [91]. As discussed above, there is some risk in pursuing FMT and morbidity and mortality from transplanted microbiota has been reported in cirrhotic patients [77].

There is some evidence supporting a vegan or plant-based Mediterranean diet to improve liver enzymes and decrease intrahepatic fat in NAFLD [93]. The data on probiotics are more robust, with early murine NAFLD models suggesting that probiotics use may decrease hepatic fat and improve liver histology, and numerous human studies demonstrating possible improvements in microbiota diversity, LPS levels and liver histology with probiotic use [94, 95]. In a meta-analysis of 21 RCTs (1252 participants) examining the effects of probiotics on NAFLD, there appeared to be a benefit of probiotics on liver enzyme levels, fibrosis, and steatosis though the data were quite heterogenous [96]. Probiotics have also been found to be beneficial in alcoholic liver disease [97, 98] and hepatic encephalopathy [99].

Finally, pathobiont removal with rifaximin has been extensively studied in liver disease. While most often used to prevent hepatic encephalopathy [100], it has also been implicated in improving outcomes in alcohol associated liver disease and NASH cirrhosis [101]. In addition, it has been studied as an agent for primary spontaneous bacterial peritonitis prophylaxis [102] and has been shown to improve portal hypertension and hemodynamics in decompensated cirrhosis [103].

## Conclusion

Overall, while the gut microbiome is known to have a role in liver disease, numerous knowledge gaps exist, particularly in PLWH. Microbiome research is rapidly growing and offers many exciting avenues for discovery. However, to fully characterize the role of the microbiome in the pathogenesis of liver disease in PLWH, further investigation (both lab-based and clinical) is required. Future research should focus on both human and animal studies and aim to analyze the mechanism and pathophysiology of microbial translocation and small molecules in liver disease and HIV. Remaining questions include the role of the microbiome in NAFLD in PLWH, how the microbiome can be used to predict liver disease progression in this population, and how diet and other environmental factors impact the gut microbiome and liver disease outcomes. As we look to the future, exploring whether the restoration of a healthy gut barrier and microbiome composition will impact liver disease and other clinical outcomes in PLWH will be a major objective, as will a deeper understanding of successful microbiome targeting interventions.

## Data Availability

Data sharing is not applicable to this article as no new data were created or analyzed in this study.
